# Oncogenic effects of RAB27B through exosome independent function in renal cell carcinoma including sunitinib-resistant

**DOI:** 10.1371/journal.pone.0232545

**Published:** 2020-05-07

**Authors:** Masafumi Tsuruda, Hirofumi Yoshino, Shunsuke Okamura, Kazuki Kuroshima, Yoichi Osako, Takashi Sakaguchi, Satoshi Sugita, Shuichi Tatarano, Masayuki Nakagawa, Hideki Enokida

**Affiliations:** Department of Urology, Graduate School of Medical and Dental Sciences, Kagoshima University, Kagoshima, Japan; University of California San Francisco, UNITED STATES

## Abstract

Exosomes are 40–100 nm nano-sized extracellular vesicles. They are released from many cell types and move into the extracellular space, thereby transferring their components to recipient cells. Exosomes are receiving increasing attention as novel structures participating in intracellular communication. RAB27B is one of the leading proteins involved in exosome secretion, and oncogenic effects have been reported in several cancers. In recent years, molecularly targeted agents typified by sunitinib are widely used for the treatment of metastatic or recurrent renal cell carcinoma (RCC). However, intrinsic or acquired resistance to sunitinib has become a major issue. The present study aimed to elucidate the role of RAB27B in RCC including sunitinib-resistant and its role in exosomes. Bioinformatic analyses revealed that high expression of RAB27B correlates with progression of RCC. The expression of RAB27B protein in RCC cell lines was significantly enhanced compared with that in normal kidney cell lines. Furthermore, RAB27B protein expression was enhanced in all of the tested sunitinib-resistant RCC cell lines compared to parental cells. Although no specific effect of RAB27B on exosomes was identified in RCC cells, loss-of-function studies demonstrated that knockdown of RAB27B suppressed cell proliferation, migration and invasive activities. Moreover, anti-tumor effects of RAB27B downregulation were also observed in sunitinib-resistant RCC cells. RNA sequence and pathway analysis suggested that the oncogenic effects of RAB27B might be associated with MAPK and VEGF signaling pathways. These results showed that RAB27B is a prognostic marker and a novel therapeutic target in sunitinib-sensitive and -resistant RCCs. Further analyses should improve our understanding of sunitinib resistance in RCC.

## Introduction

Renal cell carcinoma (RCC) constitutes approximately 90–95% of all kidney neoplasms [1], making it the seventh most common site for tumours in 2013 [2]. The incidence of RCC has risen in many countries in the last 10 years [3]. Clear cell RCC (ccRCC) is the most common subtype of kidney cancer, accounting for approximately 70–75% of cases, followed by papillary RCC (pRCC) which occurs in approximately 10–16% of cases [4]. Initially, RCC has an asymptomatic clinical course. Consequently 25–30% of all patients have metastatic disease upon diagnosis [5]. In addition, approximately 20–40% of patients who have undergone surgical resection for localized RCC suffer local recurrence or distinct metastasis during follow-up [6, 7].

Currently, molecularly targeted therapeutics such as multi-targeted receptor tyrosine kinase (RTK) or mammalian target of rapamycin (mTOR) inhibitors are generally administered for patients with metastatic or recurrent RCC. Sunitinib, a multi-targeted RTK, is one of the first-line agents for patients with advanced RCC. However, intrinsic or acquired resistance to sunitinib [8] have become a major issue for treatment. In recent years, immune checkpoint inhibitors such as anti-programmed death-1 (PD-1) antibodies and anti-cytotoxic T-lymphocyte associated antigen-4 (CTLA-4) antibodies were approved for the treatment of patients with advanced RCC. These agents cause immune checkpoint blockade and improve antitumor immune response [9]. However, phase 3 clinical trial compared PD-1 checkpoint inhibitor with mTOR inhibitor showed that the objective response rate with PD-1 checkpoint inhibitor was only 25% and the improvement of overall survival was slight [10]. Additionally, because of high cost of anti-PD-1 antibodies, there is the necessity to consider their indication from the viewpoint of cost as well as effectiveness [11]. Therefore, it is important to develop novel therapeutic strategies to overcome sunitinib resistance.

Exosomes have received increasing attention as novel methods of cell-cell communication. Extracellular vesicles (EVs), which are membranous vesicles containing cytosol from the secreting cells enclosed in a lipid bilayer, carry out important roles in substance transfer between cells [12, 13]. Exosomes represent one subtype of EV; they have a diameter of 40–100 nm and are released from many cell types into the extracellular space [14, 15]. In 2007, it was demonstrated that exosomes contain messenger RNA (mRNA) and microRNA (miRNA), and that the RNA can be shuttled from one cell to another via exosomes. In recipient cells, the mRNA shuttled by exosomes can be translated into protein, suggesting a regulatory function of the transferred RNA [16]. This report has led to the numerous studies of exosomes throughout the research community [17]. Currently, the functions of exosomes are being elucidated in oncology [18], immunology [19], neurology [20], cardiology [21] and others. In this regard, the RAB family merits attention as it represents the largest branch of the Ras-like small GTPase superfamily [22], and it controls almost all membrane trafficking processes, including vesicle budding, exosome release and docking and fusion to acceptor membranes [23, 24]. The RAB27 family, a subgroup of the RAB family, includes two distinct molecules, RAB27A and RAB27B [25]. RAB27B was originally identified in platelets [26]. Recently, it was found to be one of the leading proteins involved in exosome secretion, along with RAB27A [27–29]. In several cancers, the oncogenic effects of RAB27B have been reported, including breast cancer [30], bladder cancer [31], glioma [32] etc. However, there are no reports of its activity in renal cancer. In our previous study, we demonstrated that metabolic reprogramming occurred in sunitinib-resistant RCC cells, which were established by gavage feeding of sunitinib, resulting in the acquisition of sunitinib resistance [33]. In addition, miRNA *99a-3p* had a tumor-suppressive role through regulating ribonucleotide reductase regulatory subunit-M2 (RRM2) in sunitinib-resistant RCC cells [34]. Moreover, JQ1, an inhibitor of bromodomain containing 4 (BRD4), significantly suppressed tumor growth of sunitinib-resistant RCC cells via MYC regulation [35]. The relevance of RAB27B to sunitinib resistance has not been verified.

Accordingly, the aims of the present study were to investigate the roles of RAB27B in RCC including sunitinib-resistant and its effect on exosomes. The clinical significance of RAB27B was analyzed using The Cancer Genome Atlas (TCGA), and the expression levels of RAB27B in RCC cells and sunitinib-resistant RCC cells were evaluated. The alteration of exosome secretion by knockdown of RAB27B and cell proliferative effects of exosomes derived from RAB27B down-regulated RCC cells were examined. Loss of function assays were performed in sunitinib-sensitive and -resistant RCC cells by examining cell proliferation, migration and invasion. The mechanisms of the effects of RAB27B were investigated by RNA sequencing and pathway analysis.

## Materials and methods

### Analysis of the correlation between RAB27B and RCC

Kaplan-Meier and log-rank methods were used to analyze overall survival (OS) time using data in the OncoLnc dataset (http://www.oncolnc.org/), which contains survival data for 8,647 patients from 21 cancer studies included in TCGA. Also, OncoLnc is a useful tool for exploring survival correlations, and for downloading clinical data coupled to expression data for mRNAs, miRNAs, or long noncoding RNAs as previously described [36]. In order to evaluate the clinical relevance, a TCGA cohort database of 534 patients with ccRCC was used. Full sequencing information and clinical information were acquired using UCSC Xena (http://xena.ucsc.edu/), cBioPortal (http://www.cbioportal.org/publicportal/), and TCGA (https://tcga-data.nci.nih.gov/tcga/). The present study met the criteria for the publication guidelines provided by TCGA (http://cancergenome.nih.gov/publications/publicationguidelines).

### Human RCC cell lines and cell culture

Human RCC cells lines 786-o, A498, ACHN, Caki1 and human kidney cortex/proximal tubule epithelial cell line HK2 were purchased from the American Type Culture Collection (ATCC, Manassas, VA, USA). Routine tests for mycoplasma infection were negative. The sunitinib-resistant 786-o (SU-R-786-o) cell line was previously established by administration of sunitinib to mice which were injected 786-o cells subcutaneously [33]. SU-R-A498, SU-R-ACHN and SU-R-Caki1 were established by the same method. These cell lines were validated sunitinib resistance in xenograft assays. Parental and SU-R cells were subcutaneously injected into flanks of female nude mice (BALB/c nu/nu, 6- to 8-weeks-old, *n* = 4 for each group). After tumor formation was confirmed, we started gavage feeding of sunitinib (25mg/kg, five times a week). The tumors were harvested 20 days after injection. Comparison of the tumor volume of parental and SU-R cells were shown in S1 Fig.

Human RCC cell lines were grown in RPMI 1640 medium (Invitrogen, Carlsbad, CA, USA) supplemented with 10% fetal bovine serum (FBS) (Equitech-Bio, Inc., Kerrville, TX, USA), 50 μg/mL streptomycin, and 50 U/mL penicillin. For the experiments involving exosomes, exosome-depleted FBS (System Bioscience, LLC, Palo Alto, CA, USA) was used as a supplement in place of standard FBS. The HK2 cell line was grown in Keratinocyte Serum-Free Medium (Invitrogen) supplemented with 0.05 mg/mL bovine pituitary extract (BPE) and 5 ng/mL epidermal growth factor (EGF). These cell lines were maintained in a humidified atmosphere of 95% air/5% CO_2_ at 37˚C.

### RNA extraction and reverse transcription-quantitative polymerase chain reaction (RT-qPCR)

Total RNA was isolated using Isogen (NIPPON GENE CO., LTD., Tokyo, Japan) according to the manufacturer’s protocol, using SYBR-Green qPCR for RT-qPCR. First, 500 ng of total RNA was reverse transcribed into cDNA using the High Capacity cDNA Reverse Transcription kit (Thermo Fisher Scientific, Inc.) under the following incubation condition: 25˚C for 10 min, 37˚C for 120 min and 85˚C for 5 min. cDNA was used for q-PCR performed with the Power SYBR Green Master Mix (cat. no. 4367659; Applied Biosystems, Foster City, CA, USA) on a 7300 Real-Time PCR System (Applied Biosystems). The specificity of amplification was monitored using the dissociation curve of the amplified product. All data values were normalized with respect to glucuronidase β (*GUSB*), and the ΔΔCq method was used to calculate the fold-change. The following primers were used:

*RAB27B*, forward primer, 5′-TAGACTTTCGGGAAAAACGTGTG-3′ and reverse primer, 5′-AGAAGCTCTGTTGACTGGTGA-3′; and *GUSB*, forward primer, 5′-CGTCCCACCTAGAATCTGCT-3′ and reverse primer, 5′-TTGCTCACAAAGGTCACAGG -3′.

### Western blotting

Total protein lysates were prepared with NuPAGE LDS Sample Buffer (Invitrogen; Thermo Fisher Scientific, Inc.). Proteins were quantified with a Qubit 4 Fluorometer (Invitrogen; Thermo Fisher Scientific, Inc.). Protein lysates (20 μg) were separated on NuPAGE 4–12% Bis-Tris gels (Invitrogen; Thermo Fisher Scientific, Inc.) and transferred to polyvinylidene difluoride membranes. Immunoblotting was performed with diluted anti-RAB27B (1:1000; cat. no. 13412-1-AP; Proteintech Group, Inc., Chicago, IL, USA), anti-VEGFA (1:1000; cat. no. ab46154; abcam), anti-p38 MAPK (1:1000; cat. no. 9212; Cell Signaling Technology, Inc.), anti-phospho-p38 MAPK (1:1000; cat. no. 4511; Cell Signaling Technology, Inc.), anti-Erk1/2 (1:1000; cat. no. 4695; Cell Signaling Technology, Inc.), anti-phospho-Erk1/2 (1:2000; cat. no. 4370; Cell Signaling Technology, Inc.), anti-E-cadherin (1:1000; cat. no. #3195; Cell Signaling Technology, Inc., Danvers, MA, USA), anti-Vimentin (1:2000; cat. no. 5741; Cell Signaling Technology, Inc.), anti-N-cadherin (1:1000; cat. no. ab18203, abcam, Cambridge, UK), and anti-β-actin antibodies (1:5,000; cat. no. bs-0061R; Bioss, Beijing, China). The secondary antibodies were peroxidase-labeled anti-rabbit IgG (1 h at 25˚C; 1:5,000; cat. no. 7074S; Cell Signaling Technology, Inc.). Specific complexes were visualized using a chemiluminescence detection system (GE Healthcare Life Sciences, Little Chalfont, UK) as described previously [37]. The expression levels of these proteins were evaluated using ImageJ software (ver. 1.48; http://rsbweb.nih.gov/ij/index.html) as described previously [38, 39].

### Transfection with small interfering RNA (siRNA)

As described previously [40], human RCC cells were transfected using Lipofectamine RNAiMAX transfection reagent and Opti-MEM (both Thermo Fisher Scientific, Inc.) containing 10 nM siRNA. *RAB27B* siRNA (product ID, HSS184177 and HSS143561) or negative control siRNA (product ID, D-001810-10) (all Thermo Fisher Scientific, Inc.) to achieve loss-of-function. The knockdown efficiency of *RAB27B* siRNA was validated by confirming downregulation of *RAB27B* mRNA using RT-qPCR and RAB27B protein using western blotting.

### Isolation and quantification of exosomes

Exosomes were purified by differential centrifugation procedures, as described previously [13, 41]. Supernatants were collected from cells that had been cultured for 48 h in medium containing exosome-depleted FBS, and they were subsequently subjected to sequential centrifugation steps at 300*g* for 10 min, 2,000*g* for 10 min and 10,000*g* for 30 min to remove cell debris, dead cells and EVs other than exosomes. Supernatants were then centrifuged at 100,000*g* for 70 min at 4°C (himac CP80WX, Hitachi, Ltd., Tokyo, Japan). The pelleted exosomes were suspended in PBS and collected by ultracentrifugation at 100,000*g* for 70 min. The purified exosomes were resuspended in PBS and used in subsequent experiments.

Exosome abundance was estimated with the ExoELISA-ULTRA CD63 kit (System Bioscience, LLC.) according to the manufacturer’s protocol. This assay is a sensitive, direct Enzyme-Linked ImmunoSorbent Assay (ELISA) to quantitate exosome abundance in a given sample. The amount of exosomes is estimated by detecting CD63 on the exosome surface by specific antibody.

### Cell proliferation, migration, and invasion assays

Human RCC cells were seeded in 96-well plates with 3x10^3^ cells/well for XTT assays. After 72 h, cell proliferation was determined using a Cell Proliferation Kit II (Roche Diagnostics GmbH, Mannheim, Germany) as described previously [40].

Cell migration activity was evaluated with wound healing assays. Cells were plated in 6-well plates at 2x10^5^ cells per well, and after 48 h of transfection the cell monolayer was scraped using a P-20 micropipette tip. The initial (0 h) and residual gap lengths 24 h after wounding were calculated from photomicrographs.

Cell invasion assays were performed using modified Boyden chambers consisting of Matrigel-coated Transwell membrane filter inserts with 8-μM pores in 24-well tissue culture plates (BD Biosciences, San Jose, CA, USA). 48 h following transfection, the cells were seeded in the upper chamber of 24-well plates at 1x10^5^/well with serum-free RPMI 1640 medium. RPMI containing 10% exosome-depleted FBS in the lower chamber served as the chemoattractant, as previously described [42]. 24 h after seeding, the cells that had passed through the pores and attached to the surface of the chamber were stained by Diff-Quick (a modified Giemsa stain) (Richard Allan Scientific, San Diego, CA, USA) and counted from photomicrographs.

### RNA sequencing analysis and pathway analysis

Total RNAs from 786-o and A498 cells transfected with control siRNA or *RAB27B* siRNA were subjected to RNA sequencing, which was performed by RIKEN GENESIS CO., LTD., Tokyo, Japan. mRNA profiles were generated by NovaSeq 6000 (Illumina, Inc., San Diego, CA, USA).

Genes with significantly downregulated expression after transfection with *RAB27B* siRNA were compared with control siRNA (fold change < -1.0) and were then categorized with the Kyoto Encyclopedia of Genes and Genomes (KEGG) pathways through GeneCodis analysis (genecodis.cnb.csic.es) [43–45].

### Statistical analysis

Data are presented as means ± standard deviation of at least 3 independent experiments. The relationships between two groups were analyzed using Mann-Whitney U tests. The relationships between three or more variables and numerical values were analyzed using Bonferroni-adjusted Mann-Whitney U tests. All analyses were performed with Expert StatView software version 5.0 (SAS Institute, Inc., Cary, NC, USA). When P < 0.05, the data were accepted as showing a statistically significant difference.

## Results

### Clinical significance of *RAB27B* expression in RCC

**Fig 1:**
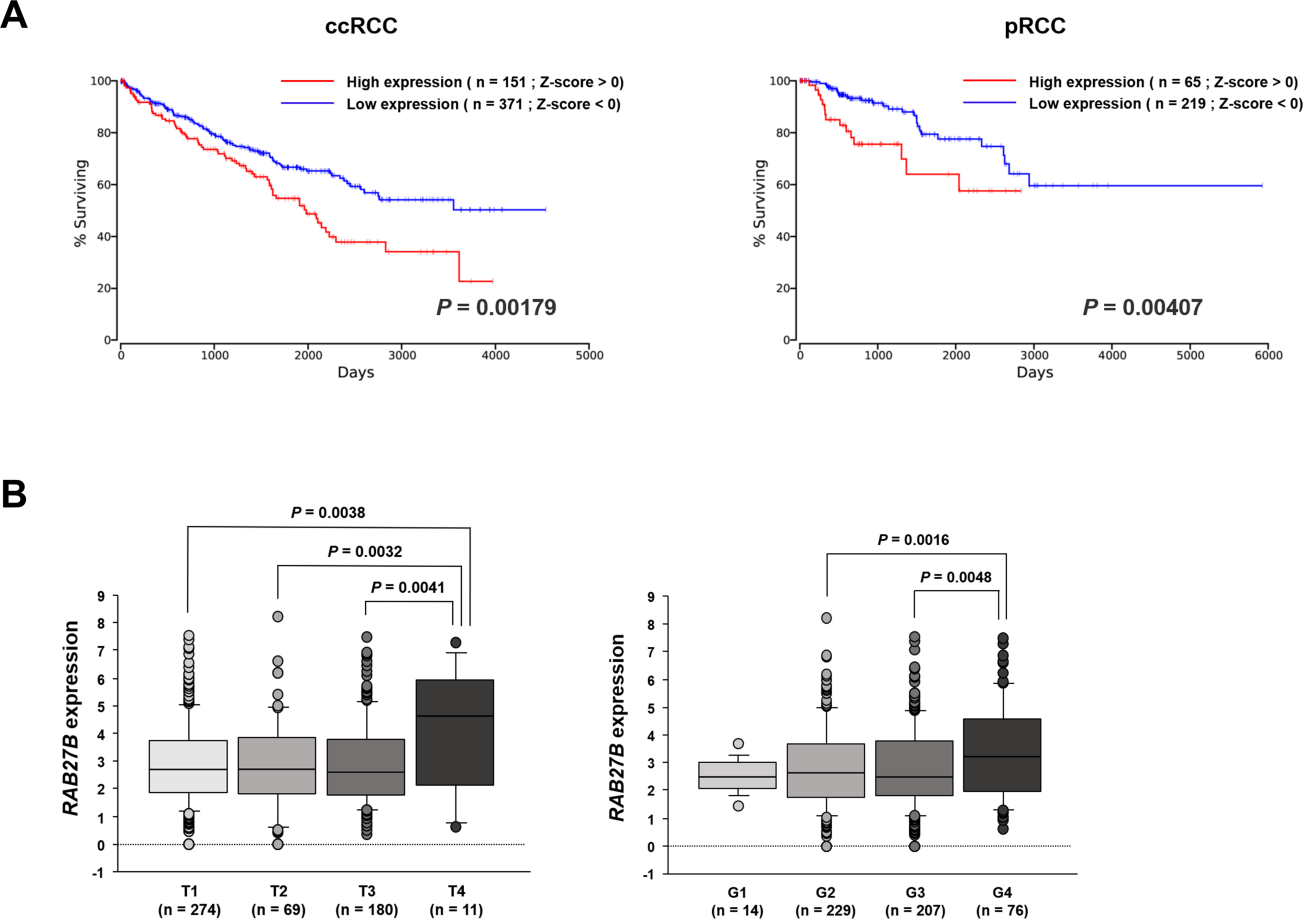
Clinical significance of *RAB27B* expression in RCC. (A) Kaplan-Meier analysis using the OncoLnc dataset revealed that the high *RAB27B* expression group (Z-score > 0) had significantly lower OS than did the low *RAB27B* expression group (Z-score < 0) in both ccRCC (right panel, P = 0.00179) and pRCC (right panel, P = 0.00407). (B) Among the ccRCC cohort of TCGA, the expression levels of *RAB27B* were significantly increased in pathological T4 category (right panel) and pathological G4 cases (left panel).

We first characterized the correlation between *RAB27B* expression and OS by performing a Kaplan-Meier analysis using the OncoLnc dataset. This analysis demonstrated that the group of patients with high expression of *RAB27B* (Z-score > 0) exhibited significantly lower OS rates compared with those in the low expression group (Z-score < 0) in both the ccRCC (*P* = 0.00179, Fig 1A, left panel) and pRCC (*P* = 0.00407, Fig 1A, right panel) cohorts. Next, we evaluated the correlations between *RAB27B* expression levels and patient clinicopathological parameters. Among the ccRCC cohort of TCGA, we found that the expression levels of *RAB27B* were significantly increased in pathological T4 category (Fig 1B, left panel) and pathological high grade G4 cases (Fig 1B, right panel). There was no significant difference of *RAB27B* expression between normal tissues and ccRCCs/pRCCs (S2 Fig).

**Fig 2:**
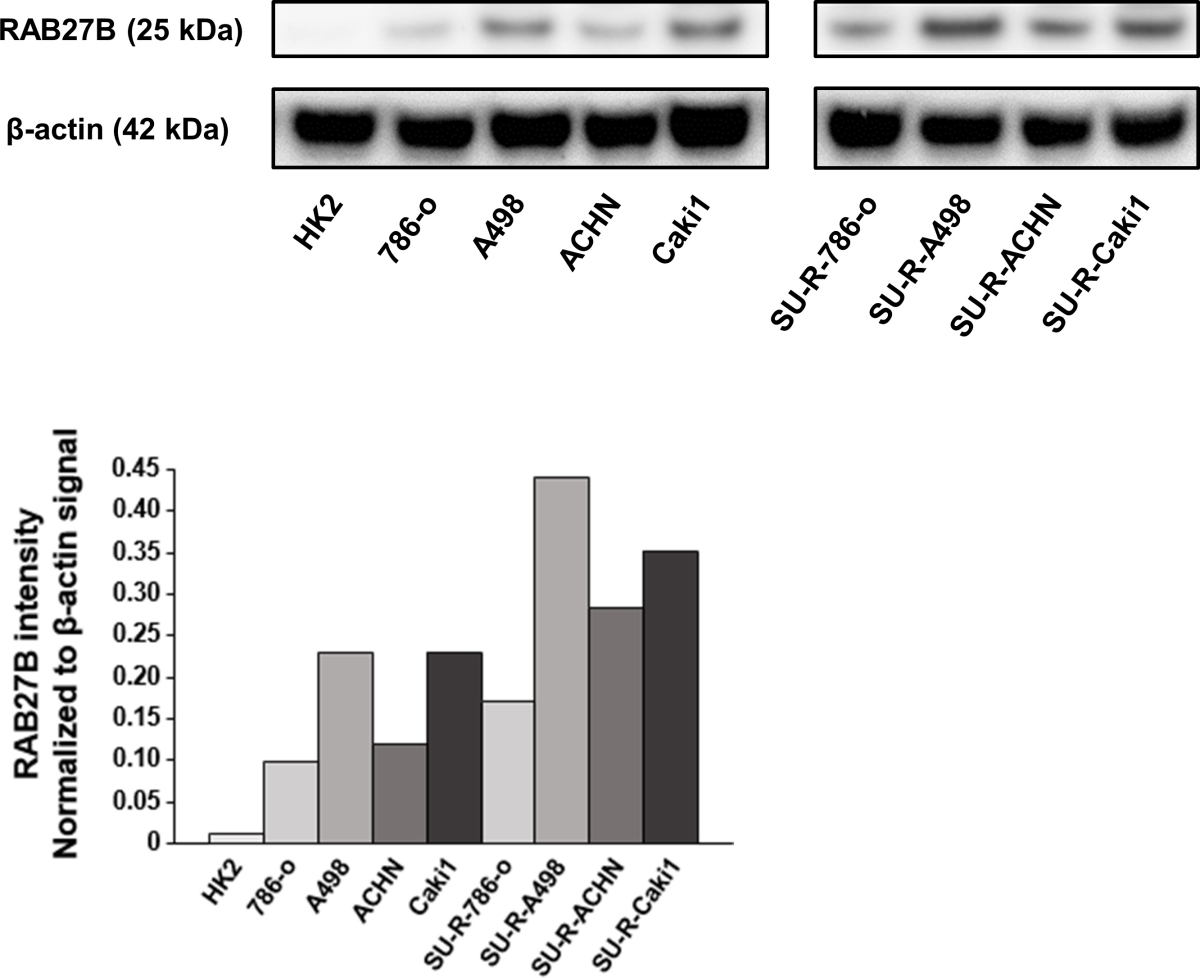
Analysis of RAB27B protein expression in RCC cells. RAB27B protein expression levels in RCC cell lines, SU-R-RCC cell lines and HK2 cells were determined by Western blotting and densitometric analyses. The expression levels of RAB27B protein in RCC cell lines were elevated in comparison with that in HK2 cells. Furthermore, in all of the sunitinib-resistant RCC cell lines, the expression of RAB27B protein was enhanced compared to their parent cell lines. β-actin was used as a loading control.

Then, RAB27B protein expression levels in RCC cell lines, sunitinib-resistant RCC cell lines and HK2 cells were evaluated by Western blot analyses. The expression levels of RAB27B protein in RCC cell lines were elevated in comparison with the levels in HK2. Furthermore, it was revealed that the expression of RAB27B protein in all of the sunitinib-resistant RCC cell lines was enhanced compared to their parent cell lines (Fig 2).

### Functional investigation of RAB27B for exosomes in RCC

**Fig 3:**
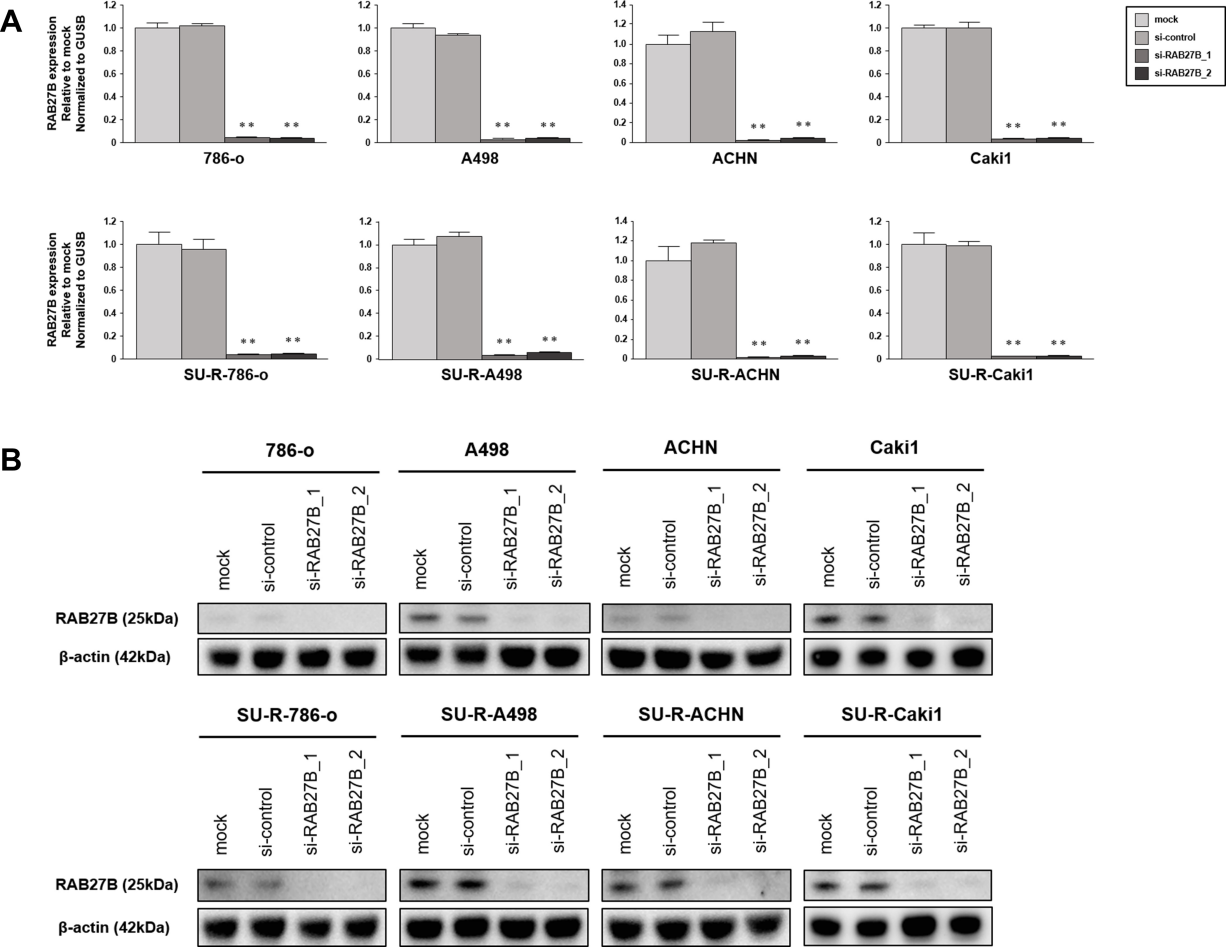
Analyses of knockdown efficiency of si-*RAB27B*. (A) *RAB27B* mRNA expressions were measured by RT-qPCR to validate the knockdown efficiency of si-*RAB27B*. The expression levels of *RAB27B* mRNA were effectively downregulated in the si-*RAB27B*-transfected RCC cells (**P < 0.001). (B) The expression of RAB27B protein were measured by western blot analyses. RAB27B protein levels were also downregulated in the cells transfected with si-*RAB27B*.

To analyze the function of RAB27B for exosomes, we employed A498 cells because of their high expression level of RAB27B. First, we investigated whether knockdown of RAB27B reduced the secretion of exosomes. RT-qPCR analyses indicated effective downregulation of *RAB27B* mRNA in the si-*RAB27B*-tansfected RCC cells (Fig 3A). Western blot analyses revealed that RAB27B protein levels were also downregulated in the cells transfected with si-*RAB27B* (Fig 3B).

A498 cells transfected with si-control or si-*RAB27B* were cultured in RPMI 1640 medium supplemented with exosome-depleted FBS. Cell culture conditioned media were collected 48 h after transfection, and exosomes in the medium were isolated by ultracentrifugation and resuspended in PBS. We measured the amount of exosomes isolated from 40 mL of conditioned medium with the ExoELISA-ULTRA CD63 kit. Although the quantity of exosomes in the medium of si-*RAB27B*-transfected cells tended to be less than that of si-control-transfected cells, there was no statistically significant difference between them (S3A Fig).

We evaluated the effect of exosomes on cell proliferation with the XTT assay. We initially checked the effect of A498 exosomes on their own proliferation. A498 exosomes were added into the medium of A498 cells seeded in 96-well plates, and cell proliferation was determined after 72 h. XTT assays revealed that exosomes derived from si-*RAB27B*-transfected cells had no significant effect on cell proliferation compared to those from mock and si-control transfectants (S3B Fig, left panel). Subsequently, we examined the effect on the proliferation of cells other than the cells from which the exosomes were derived. We obtained the same results in the experiment when we added A498 exosomes to 786-o cells (S3B Fig, middle panel) and SU-R-786-o cells (S3B Fig, right panel). Thus, no specific effect of RAB27B on exosomal function was revealed in RCC cells in regard to both quantity and function.

### Oncogenic effects of RAB27B in RCC cells

**Fig 4:**
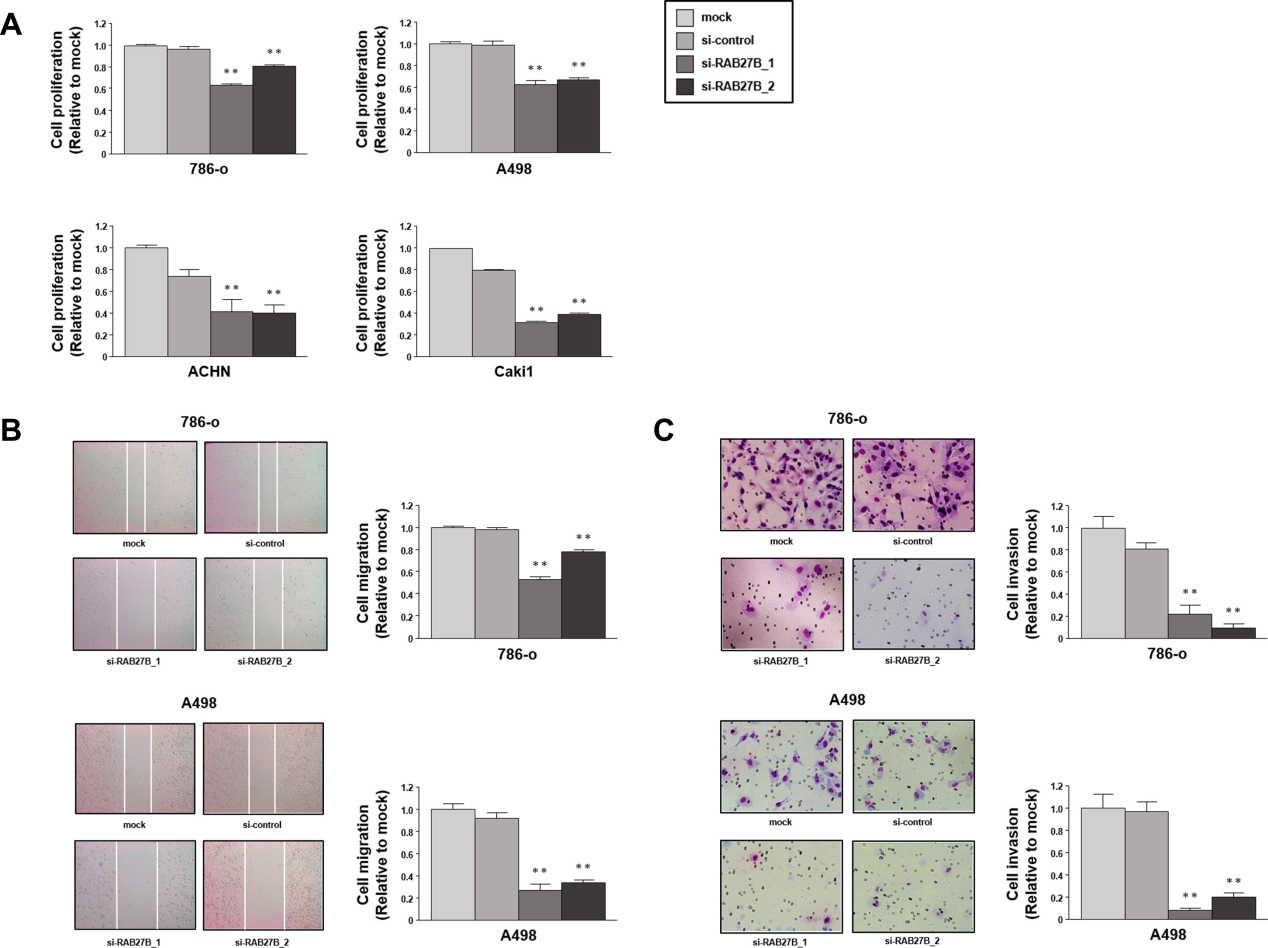
Oncogenic effects of RAB27B in RCC cells. (A) Cell proliferation was examined using XTT assays in cells after RAB27B knockdown, revealing a significant inhibition compared with the control groups. (B) Wound healing assays revealed suppressed migratory activity in si-*RAB27B* transfected 786-o and A498 cells. (C) Matrigel invasion assays indicated that the number of invading cells was significantly decreased by RAB27B knockdown in 786-o and A498 cells. (**, P < 0.001).

Loss-of-function studies using si-*RAB27B* were conducted to investigate the functional role of RAB27B in RCC cells. XTT assays with 786-o, A498, ACHN and Caki1 cells demonstrated that cell proliferation was inhibited in si-*RAB27B*-transfectants in comparison with that in the mock- or si-control-transfectants (Fig 4A). Wound healing assays revealed that cell migration activity was also inhibited in si-*RAB27B*-transfected 786-o and A498 cells (Fig 4B). Similarly, Matrigel invasion assays revealed that the number of invading cells was significantly decreased in si-*RAB27B*-transfected 786-o and A498 cells (Fig 4C). From the above, the oncogenic effects of RAB27B in RCC cells were demonstrated.

### Oncogenic effects of RAB27B in sunitinib-resistant RCC cells

**Fig 5:**
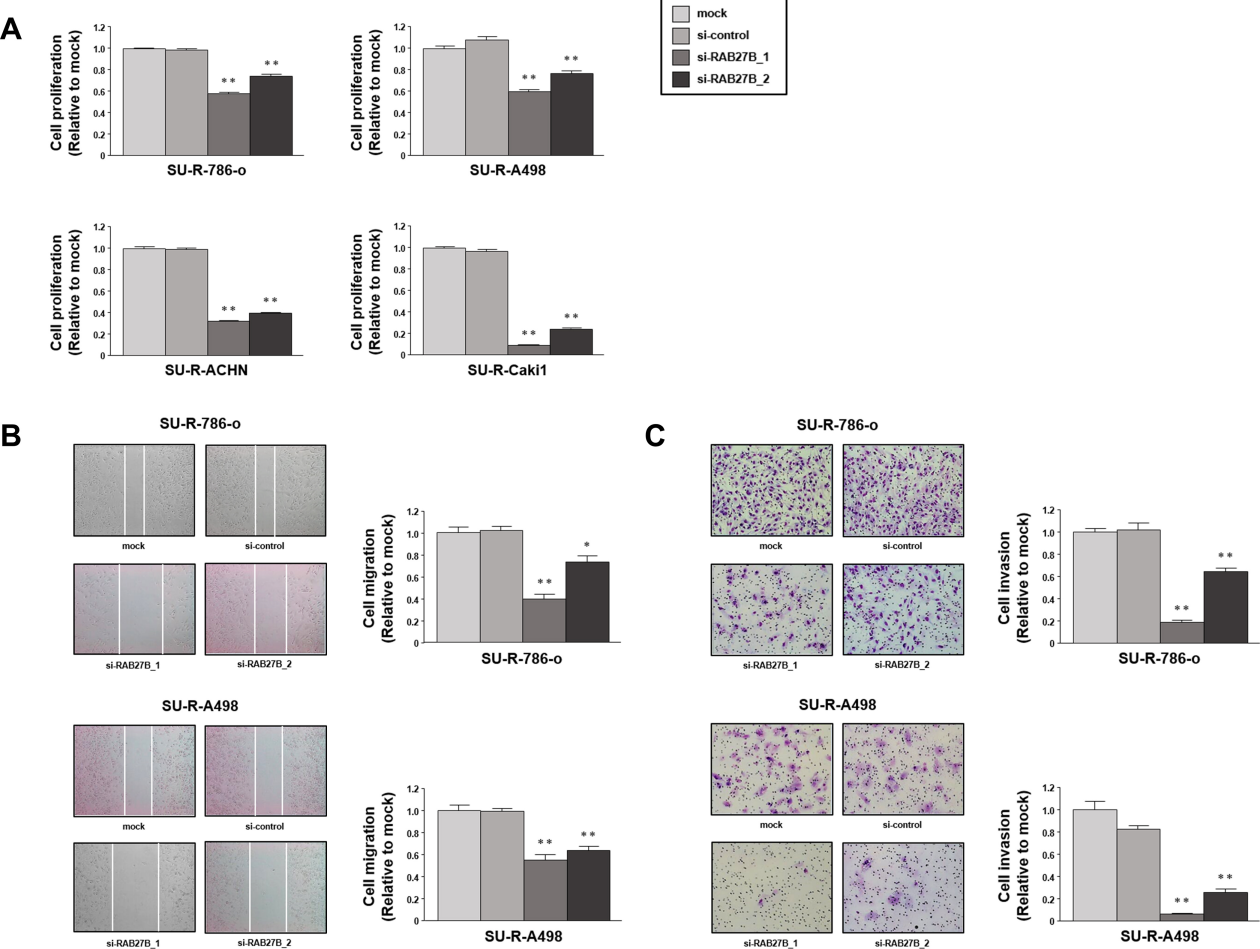
Oncogenic effects of RAB27B in sunitinib-resistant RCC cells. (A) Cell proliferation assessed by XTT assays was reduced in si-*RAB27B*-transfected sunitinib-resistant RCC cells. (B) Cell migration activity was suppressed by RAB27B knockdown in SU-R-786-o and SU-R-A498 cells. (C) Matrigel invasion activity was significantly decreased by si-*RNA27B* transfection of SU-R-786-o and SU-R-A498 cells. (**, P < 0.001; *, P < 0.01).

Subsequently, we investigated the functional role of RAB27B in sunitinib-resistant RCC cells. Loss-of-function assays by transfection with si-*RAB27B* were performed in sunitinib-resistant RCC cells as well as parental cells. Cell proliferation assessed by XTT assays was reduced in si-*RAB27B*-transfected SU-R-786-o, SU-R-A498, SU-R-ACHN and SU-R-Caki1 cells (Fig 5A). Cell migration activity was suppressed in si-*RAB27B*-transfected SU-R-786-o and SU-R-A498 cells (Fig 5B). Cell invasion activity was also suppressed by si-*RNA27B* transfection in SU-R-786-o and SU-R-A498 cells (Fig 5C). These results indicated that high expression of RAB27B was associated with oncogenic effects even in sunitinib-resistant RCC cells.

### Analysis of the mechanism of oncogenic effects of RAB27B

In order to investigate the mechanism of oncogenic effects of RAB27B, RNA sequence and pathway analyses were performed (DRA009693: https://ddbj.nig.ac.jp/DRASearch/submission?acc=DRA009693). A heatmap of RNA sequencing data was shown in S4 Fig. Based on the RNA sequencing data, we found 1069 genes downregulated in 786-o cells transfected with si-*RAB27B*, and 436 genes downregulated in A498 cells transfected with si-*RAB27B* compared with each cell type transfected with si-control. Among the 185 genes common to both, the most downregulated 20 genes are displayed in Table 1.

**Table 1:** Downregulated genes in renal cancer cells transfected with si-*RAB27B*.

		fold change		
Gene symbol	Description	786-o	A498	average
*TMEM30A*	transmembrane protein 30A	-3.042	-3.459	-3.250
*RPPH1*	ribonuclease P RNA component H1	-4.272	-1.964	-3.118
*PMCH*	pro-melanin-concentrating hormone	-2.029	-4.044	-3.037
*ATP6AP2*	ATPase, H+ transporting, lysosomal accessory protein 2	-2.950	-2.889	-2.919
*SLC16A1*	solute carrier family 16 (monocarboxylate transporter), member 1	-2.627	-2.903	-2.765
*SEC23A*	Sec23 homolog A (S. cerevisiae)	-2.568	-2.713	-2.640
*NXT2*	nuclear transport factor 2 like export factor 2	-2.293	-2.954	-2.624
*TGFBR1*	transforming growth factor, beta receptor 1	-2.318	-2.850	-2.584
*IL17D*	interleukin 17D	-3.040	-2.123	-2.581
*C15orf48*	chromosome 15 open reading frame 48	-2.771	-2.361	-2.566
*CEP55*	centrosomal protein 55kDa	-2.412	-2.620	-2.516
*GSKIP*	GSK3B interacting protein	-2.307	-2.607	-2.457
*MAL2*	mal, T-cell differentiation protein 2	-2.743	-2.117	-2.430
*PPP3CA*	protein phosphatase 3, catalytic subunit, alpha isozyme	-2.096	-2.761	-2.428
*SNHG8*	small nucleolar RNA host gene 8	-3.271	-1.582	-2.427
*RARRES2*	retinoic acid receptor responder (tazarotene induced) 2	-1.175	-3.677	-2.426
*CYP1B1*	cytochrome P450, family 1, subfamily B, polypeptide 1	-2.341	-2.491	-2.416
*UBLCP1*	ubiquitin-like domain containing CTD phosphatase 1	-2.256	-2.574	-2.415
*LBR*	lamin B receptor	-2.100	-2.715	-2.407
*SLC25A15*	solute carrier family 25 (mitochondrial carrier; ornithine transporter) member 15	-2.265	-2.505	-2.385

Next, we used GeneCodis analysis to categorize the si-*RAB27B-*downregulated genes into KEGG pathways. The results revealed that these genes were contained in 24 pathways, including the Vascular Endothelial Growth Factor (VEGF) signaling pathway, which is the main target of sunitinib, and Mitogen-activated Protein Kinase (MAPK) signaling pathway, a representative oncogenic pathway. These pathways were listed in descending order of corrected P-values in Table 2. Therefore, we evaluated the expression levels of several proteins involved in VEGF and MAPK signaling pathway by western blot analyses in 786-o, A498, SU-R-786-o and SU-R-A498 cells. Even though no common alterations of the expression pattern of these proteins after si-*RAB27B* transfection in the RCC cells, Erk1/2 or p38 MAPK protein was faintly decreased in 786-O, A498, or SU-R-786-O after si-RAB27B transfection. (S5 Fig).

**Table 2:** Downregulated KEGG pathways in renal cancer cells transfected with si-*RAB27B*.

KEGG ID	Annotations	Number of genes	Corrected P-value	Genes
5016	Huntington's disease	18	0.001028	*NDUFA2*,*NDUFS8*,*POLR2G*,*ATP5D*,*AP2S1*, *POLR2L*,*PPID*,*NDUFB10*,*COX5B*,*DCTN4*, *NDUFB3*,*SOD2*,*NDUFA1*,*POLR2J3*, *NDUFA11*,*POLR2I*,*NDUFS7*,*COX7A2L*
5012	Parkinson's disease	15	0.001299	*NDUFA2*,*NDUFS8*,*ATP5D*,*PPID*,*UBE2L6*, *NDUFB10*,*COX5B*,*UBE2J2*,*NDUFB3*, *NDUFA1*,*UCHL1*,*NDUFA11*,*NDUFS7*, *COX7A2L*,*UBB*
280	Valine, leucine and isoleucine degradation	9	0.001448	*ACAT2*,*ALDH1B1*,*ACADM*,*MCCC1*, *ALDH6A1*,*ABAT*,*HMGCL*,*ACADSB*,*AOX1*
4120	Ubiquitin mediated proteolysis	14	0.004448	*UBE2C*,*DDB2*,*UBE2L6*,*ANAPC13*,*UBE2J2*, *BIRC3*,*SYVN1*,*ERCC8*,*ANAPC11*,*RBX1*, *UBE2S*,*TCEB2*,*RNF7*,*STUB1*
240	Pyrimidine metabolism	11	0.006179	*NT5C*,*POLR2G*,*POLR3K*,*POLR2L*,*ITPA*, *DTYMK*,*POLD4*,*POLR2J3*,*TK1*,*POLR3E*, *POLR2I*
3020	RNA polymerase	6	0.006328	*POLR2G*,*POLR3K*,*POLR2L*,*POLR2J3*, *POLR3E*,*POLR2I*
3008	Ribosome biogenesis in eukaryotes	9	0.012605	*FCF1*,*SBDS*,*UTP18*,*NOP58*,*NXT2*,*GTPBP4*, *POP5*,*GNL3*,*RPP25*
4114	Oocyte meiosis	11	0.014851	*ESPL1*,*CCNB2*,*PTTG1*,*ANAPC13*,*RPS6KA2*, *MAPK12*,*PLK1*,*ANAPC11*,*PPP3CA*,*RBX1*, *PPP1CB*
5010	Alzheimer's disease	14	0.015760	*BAD*,*CASP7*,*NDUFA2*,*NDUFS8*,*ATP5D*, *NDUFB10*,*COX5B*,*CAPN2*,*NDUFB3*,*PPP3CA*, *NDUFA1*,*NDUFA11*,*NDUFS7*,*COX7A2L*
190	Oxidative phosphorylation	12	0.015966	*NDUFA2*,*NDUFS8*,*ATP5D*,*NDUFB10*, *COX5B*,*NDUFB3*,*ATP6V0C*,*NDUFA1*, *ATP6V1E2*,*NDUFA11*,*NDUFS7*,*COX7A2L*
900	Terpenoid backbone biosynthesis	4	0.017628	*ACAT2*,*MVD*,*IDI1*,*IDI2*
4510	Focal adhesion	15	0.023570	*BAD*,*RELN*,*PGF*,*PDGFC*,*RAC2*,*BIRC3*, *CAPN2*,*CAV1*,*RAC3*,*VASP*,*ITGAV*,*FYN*, *CTNNB1*,*MYL5*,*PPP1CB*
4110	Cell cycle	11	0.023941	*ESPL1*,*CCNB2*,*PTTG1*,*ANAPC13*,*SFN*, *STAG2*,*PLK1*,*ANAPC11*,*CCNA1*,*RBX1*, *CDKN1C*
380	Tryptophan metabolism	6	0.024668	*ACAT2*,*CAT*,*ALDH1B1*,*KMO*,*CYP1B1*,*AOX1*
4370	VEGF signaling pathway	8	0.031072	*BAD*,*RAC2*,*PLA2G6*,*MAPK12*,*RAC3*, *PPP3CA*,*KRAS*,*HSPB1*
5210	Colorectal cancer	7	0.032788	*BAD*,*TGFBR1*,*RAC2*,*RAC3*,*KRAS*,*APPL1*, *CTNNB1*
4122	Sulfur relay system	3	0.033906	*MPST*,*TST*,*CTU1*
640	Propanoate metabolism	5	0.034257	*ACAT2*,*ALDH1B1*,*ACADM*,*ALDH6A1*,*ABAT*
4670	Leukocyte transendothelial migration	10	0.036059	*CLDN2*,*CTNNA1*,*RAC2*,*CYBA*,*MAPK12*, *VASP*,*NCF2*,*CTNNB1*,*MYL5*,*CLDN14*
4010	MAPK signaling pathway	17	0.038571	*CACNA2D1*,*RPS6KA5*,*NGF*,*IKBKG*, *MAP3K13*,*TGFBR1*,*RAC2*,*PLA2G6*, *RPS6KA2*,*GNG12*,*MAPK12*,*RAC3*,*RRAS2*, *PPP3CA*,*KRAS*,*HSPB1*,*DUSP14*
4520	Adherens junction	7	0.049024	*CTNNA1*,*TGFBR1*,*PTPRM*,*RAC2*,*RAC3*, *FYN*,*CTNNB1*
5200	Pathways in cancer	19	0.049311	*BAD*,*CTNNA1*,*PGF*,*IKBKG*,*TGFBR1*,*RAC2*, *TPM3*,*BIRC3*,*WNT5B*,*RAC3*,*ITGAV*,*KRAS*, *APPL1*,*CEBPA*,*CCNA1*,*CTNNB1*,*RBX1*, *FLT3LG*,*TCEB2*
3010	Ribosome	8	0.049395	*RPL13*,*RPS19*,*RPL5*,*RPL18*,*RPS13*, *RPL36AL*,*RPS29*,*RPS20*
4914	Progesterone-mediated oocyte maturation	8	0.049395	*CCNB2*,*ANAPC13*,*RPS6KA2*,*MAPK12*, *PLK1*,*ANAPC11*,*KRAS*,*CCNA1*

KEGG, Kyoto Encyclopedia of Genes and Genomes.

## Discussion

Extracellular vesicles are classified into exosomes, microvesicles and apoptotic bodies based on their size, components, and biogenic mechanism [46]. Exosomes are derived from intracellular vesicles called multivesicular endosomes (MVEs). Endosomes are formed by endocytosis, after which a large number of intraluminal vesicles (ILVs) bud into endosomes, becoming MVEs. MVEs can directly fuse with the plasma membrane, which leads to release of the ILVs to the extracellular environment as exosomes, where they function in a multitude of intercellular signaling processes [12, 14, 47]. RAB27A and RAB27B are thought to function in MVEs’ docking to the plasma membrane [15, 29]. Furthermore, RAB27B has been suggested to be involved in the transfer of MVEs to the plasma membrane [29, 48]. Contrary to expectations, suppression of exosome release was not observed in cells transfected with si-*RAB27B* in the present study. Since exosome secretion has been reported to increase in environments that are not suitable for cell survival, including chemotherapeutic treatment, hypoxia, heat stress, etc. [49], it is possible that the tumor-suppressive effects of si-*RAB27B* acted to promote exosome secretion. Further studies are necessary to elucidate the mechanism underlying RAB27B and exosome secretion in RCC.

Exosomes have a range of different roles in cancer and they can be used as diagnostic markers and predict therapeutic responses. They may also constitute targets in therapeutic applications. Chen et al. reported that PD-ligand 1 (PD-L1) on exosome surfaces released from metastatic melanoma cells suppressed tumor immunity, and circulating exosomal PD-L1 can be a response predictor of anti-PD-1 therapy [50]. Kamerkar et al. demonstrated that exosomes derived from mesenchymal cells artificially incorporated siRNA or short hairpin (sh) RNA specific for oncogenic *KRAS* suppressed cancer in a plurality of mouse models of pancreatic cancer [51]. In renal cancer, differential protein profiling in urinary exosomes [52] and miRNAs contained in serum exosomes [53, 54] represent potential diagnostic markers. In addition, exosomes released from renal cancer stem cells contribute to triggering angiogenesis at premetastatic niches in the lung [55]. Further on, exosomes containing carbonic anhydrase 9 (CA9), a cellular response to hypoxia, were released from hypoxic RCC cells, and they are suggested to enhance angiogenesis in the microenvironment, thereby contributing to cancer progression [56]. Moreover, exosomes derived from RCC cells induce apoptosis of Jurkat T cell, suggesting they may contribute to immune evasion of tumors [57].

In the present study, the relevance of RAB27B to exosome secretion was not identified. Nevertheless, knockdown of RAB27B showed inhibitory effects of cell proliferation as well as cell migration and invasion. Therefore, we hypothesized that another functional role of RAB27B might be involved in epithelial-mesenchymal transition (EMT). Even though the western blot analyses of several representative EMT markers showed no common alterations in the RCC cells, the protein expression levels of Vimentin and N-cadherin were faintly decreased in 786-O or SU-R-A498 cells after si-RAB27B transfection (S6 Fig), suggesting that RAB27B might partially contributed to the EMT process in RCC as was previously reported in breast cancer [58].

As well as our demonstration, the oncogenic effects of RAB27B have been reported in several types of cancer. RAB27B has been shown to regulate invasive growth *in vitro* and *in vivo* in estrogen receptor (ER)-positive breast cancer cell lines [30] and to be involved in osteosarcoma cell migration and invasion [59]. Furthermore, it was suggested that disposal of tumor-suppressive miRNA via exosome release through the function of RAB27B is associated with acquisition of metastatic properties in bladder cancer [31]. According to our analysis, the expression level of RAB27B was associated with poor prognosis in RCCs. It has been demonstrated that high expression of RAB27B is correlated with poor prognosis in hepatocellular carcinoma [60], colorectal cancer [61] and ovarian cancer [62], consistent with our results. Although hypomethylated RAB27B is reported to be a progression-associated prognostic biomarker in glioma [32], the regulatory mechanisms of RAB27B expression remain to be defined. Thus, further research on the mechanisms is important to better understand these processes.

In terms of acquisition of drug resistance, a number of studies have demonstrated the contribution of exosomes [63]. For instance, there are several reports indicating a relationship between chemoresistance and exosomes in prostatic cancer [64–66]. In neuroblastoma [67] and ovarian cancer, [68] exosomal transfer of *miR21* between cancer cells and stromal cells was indicated to contributes to development of chemoresistance. Recently, it was also demonstrated that ALK in the exosomes secreted by BRAF inhibitor-resistant melanoma cells transferred drug resistance through activation of the MAPK signaling pathway in recipient cells [69]. Furthermore, in renal cancer, Qu et al. showed that long noncoding RNA transmitted by exosomes promoted acquisition of sunitinib resistance [70]. With respect to RAB27B, metabolic reprogramming mediated by RAB27B was verified to induce doxorubicin resistance in breast cancer cells [71], and it was found that RAB27B is involved in chemoresistance to cisplatin in pancreatic cancer [72]. Moreover, exosome transfer from stroma to cancer cells regulated by stromal RAB27B is involved in therapeutic resistance in breast cancer [73]. In our study, RAB27B showed oncogenic effects in RCC cell lines with sunitinib resistance. Since the expression of RAB27B protein in all of the sunitinib-resistant RCC cell lines was enhanced compared to the parental cells, RAB27B may have some involvement in sunitinib resistance acquisition.

In order to investigate the mechanism underlying the oncogenic effects of RAB27B and its relevance to sunitinib resistance, pathway analysis was performed. GeneCodis analysis demonstrated that the VEGF signaling pathway and the MAPK signaling pathway were downregulated in RAB27B-knockdown cells. VEGF plays an important role in pathological angiogenesis associated with tumor growth [74], and also act as an autocrine growth factor [75]. VEGF have long been regarded as a promising therapeutic target in RCC [76], and is a major target molecule of sunitinib. Ishibashi et al. reported that tyrosine kinase inhibitors (TKIs) treatment to 786-o cells enhanced the expression of IL-6 and VEGF, and suggested that combination therapy of IL-6 inhibitor and TKIs may overcome TKI resistance [77]. MAPK pathway, which play essential roles in cell differentiation, proliferation and survival, also has been reported to be possible therapeutic target in RCC [78]. In addition, Gao et al. demonstrated that treatment targeting the ERK/MAPK pathway suppressed sunitinib resistance [79]. In ER-negative breast cancer, RAB27B-mediated modulation of β-catenin and VEGF was reported [80]. Further, in pancreatic cancer, EVs released by upregulated RAB27B activated p38 MAPK [81]. From the above, RAB27B may be associated with activated VEGF and MAPK signaling. Although knockdown of RAB27B showed no common alterations of the expression pattern of VEGF- or MAPK-related proteins in the western blot analyses, Erk1/2 or p38 MAPK protein was faintly decreased in 786-O, A498, or SU-R-786-O after si-RAB27B transfection (S5 Fig), suggesting that RAB27B might partially contributed to accelerating MAPK pathway in RCC. Interestingly, pathway analysis also showed an association between RAB27B and neurological diseases such as Huntington's disease, Parkinson's disease and Alzheimer's disease (AD). In fact, there are several reports showing the involvement of exosomes in these diseases [82–84]. Additionally, the upregulation of some synaptic GTPases, including RAB27B, was detected in tissues from patients with higher degrees of AD, and aberrant synaptic trafficking was suggested to modulate the progression of AD [85].

In conclusion, the present study investigated the association between RAB27B, an exosome secretory protein, and RCC. Although specific effects of RAB27B on exosomes were not identified, the oncogenic effects of RAB27B in RCC cell lines were demonstrated. Furthermore, the oncogenic effects of RAB27B were also demonstrated in sunitinib-resistant RCC cell lines. RAB27B may be a novel therapeutic target for sunitinib-sensitive and -resistant RCC. In addition, further studies may provide new insights into improving our understanding of sunitinib resistance in RCC.

## Supporting information

S1 Fig Establishment of sunitinib-resistant A498, ACHN and Caki1 cells. SU-R-A498, SU-R-ACHN and SU-R-Caki1 cells were validated their sunitinib resistance in xenograft assays under sunitinib treatment. The tumor volumes of SU-R cells were significantly greater than those of parental cells. (*, P < 0.05).
(TIF)

S2 Fig Comparison of *RAB27B* expression between normal and RCC cohort of TCGA. There was not significant difference of *RAB27B* expression both in ccRCC and pRCC.
(TIF)

S3 Fig Investigation of the function of RAB27B in exosomes in RCC. (A) Cell culture-conditioned medium was used for exosome isolation by ultracentrifugation and the amounts of exosomes were estimated by direct ELISA to CD63 on the surface of exosomes. There was no statistically significant difference between the accumulation of exosomes in si-control transfected cell culture medium and that in si-*RAB27B* transfected cell culture medium. (B) Exosomes derived from A498 cells were added to A498 cells (left panel), 786-o cells (middle panel) and SU-R-786-o cells (right panel). The XTT assay was performed 72 h after adding each batch of exosomes. Exosomes derived from si-*RAB27B*-transfected cells had no significant effect on cell proliferation compared to those from mock and si-control transfectant. n.s.; not significant.
(TIF)

S4 Fig Heatmap of RNA sequencing data. (TIF)

S5 Fig Evaluation of the expression levels of proteins involved in MAPK and VEGF signaling pathway. There was no certain tendency in the alteration of the expression pattern of VEGF protein after transfection of si-*RAB27B*. However, Erk1/2 and p38 MAPK proteins were somewhat downregulated by RAB27B knockdown in a few cell lines.
(TIF)

S6 Fig Western blot analyses of EMT markers. The protein expression levels of Vimentin and N-cadherin were somewhat decreased in several cell lines after knockdown of RAB27B.
(TIF)

S1 Dataset (XLSX)
