# Supplementary figures and images for: Oncogenic effects of RAB27B through exosome independent function in renal cell carcinoma including sunitinib-resistant
Source: PLoS One. 2020 May 7;15(5):e0232545. doi: 10.1371/journal.pone.0232545 (PMC7205224; doi:10.1371/journal.pone.0232545)

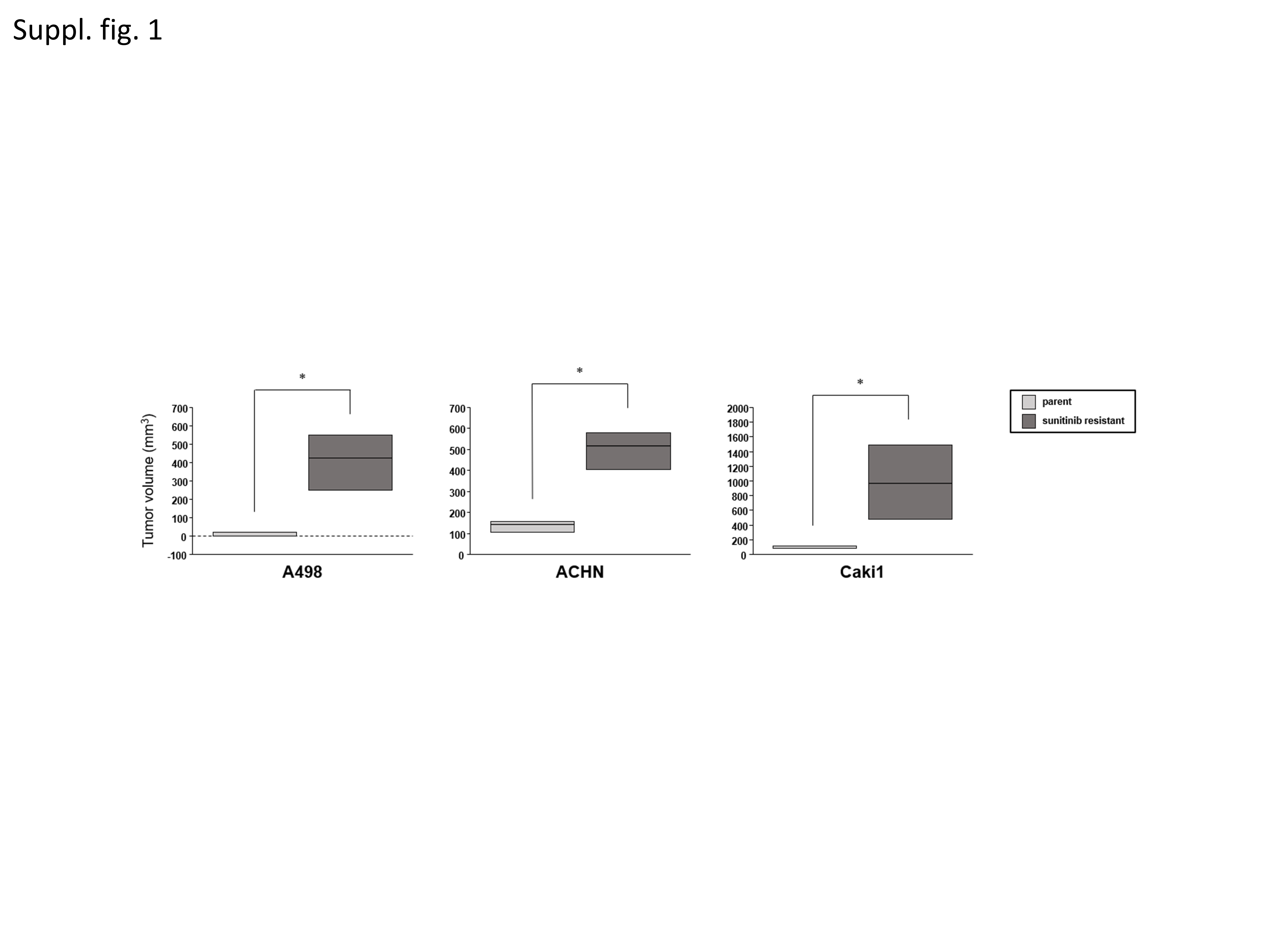

Supplement: S1 Fig — SU-R-A498, SU-R-ACHN and SU-R-Caki1 cells were validated their sunitinib resistance in xenograft assays under sunitinib treatment. The tumor volumes of SU-R cells were significantly greater than those of parental cells. (*, P < 0.05). (TIF) [file pone.0232545.s001.tif]

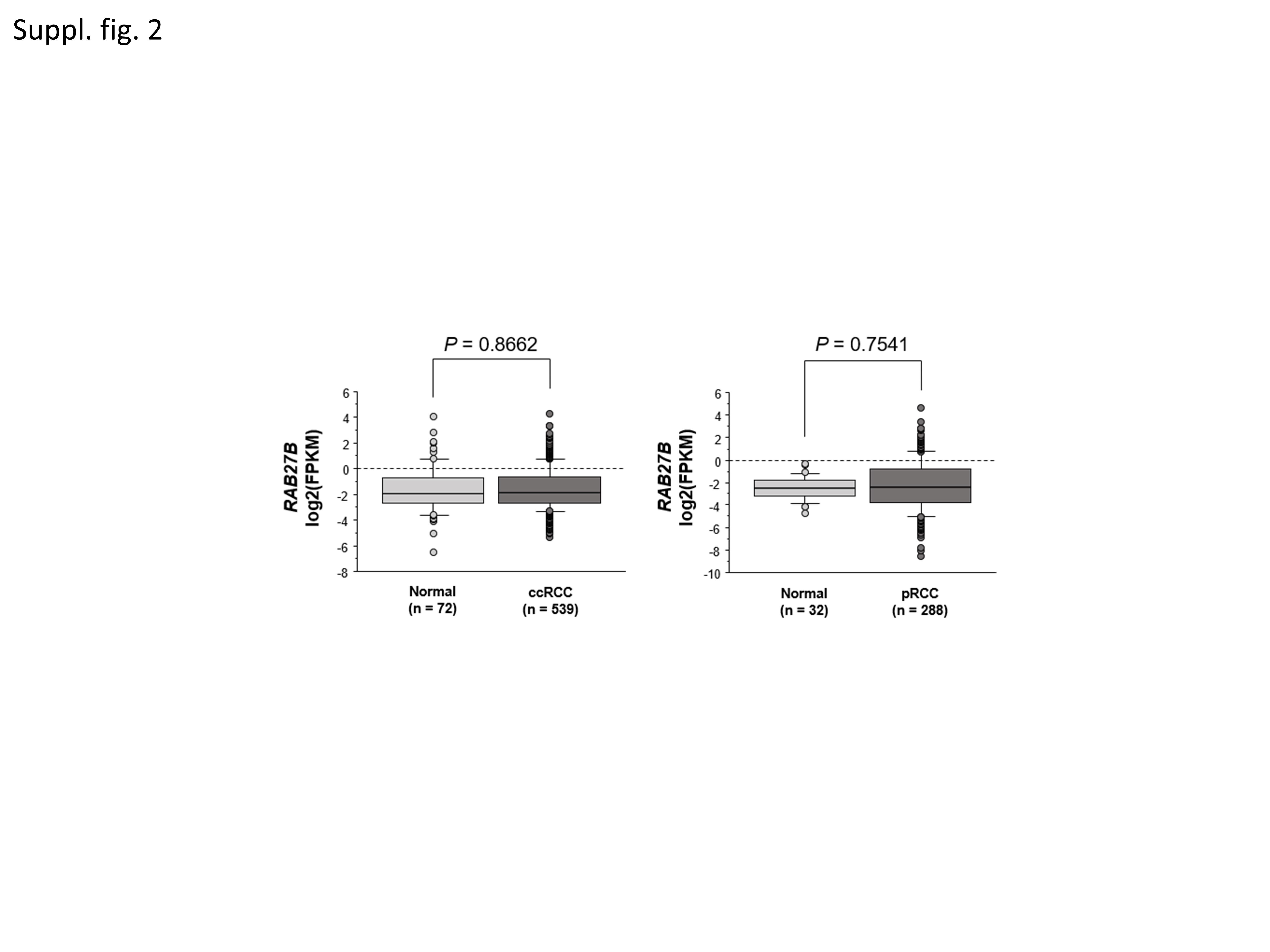

Supplement: S2 Fig — There was not significant difference of RAB27B expression both in ccRCC and pRCC. (TIF) [file pone.0232545.s002.tif]

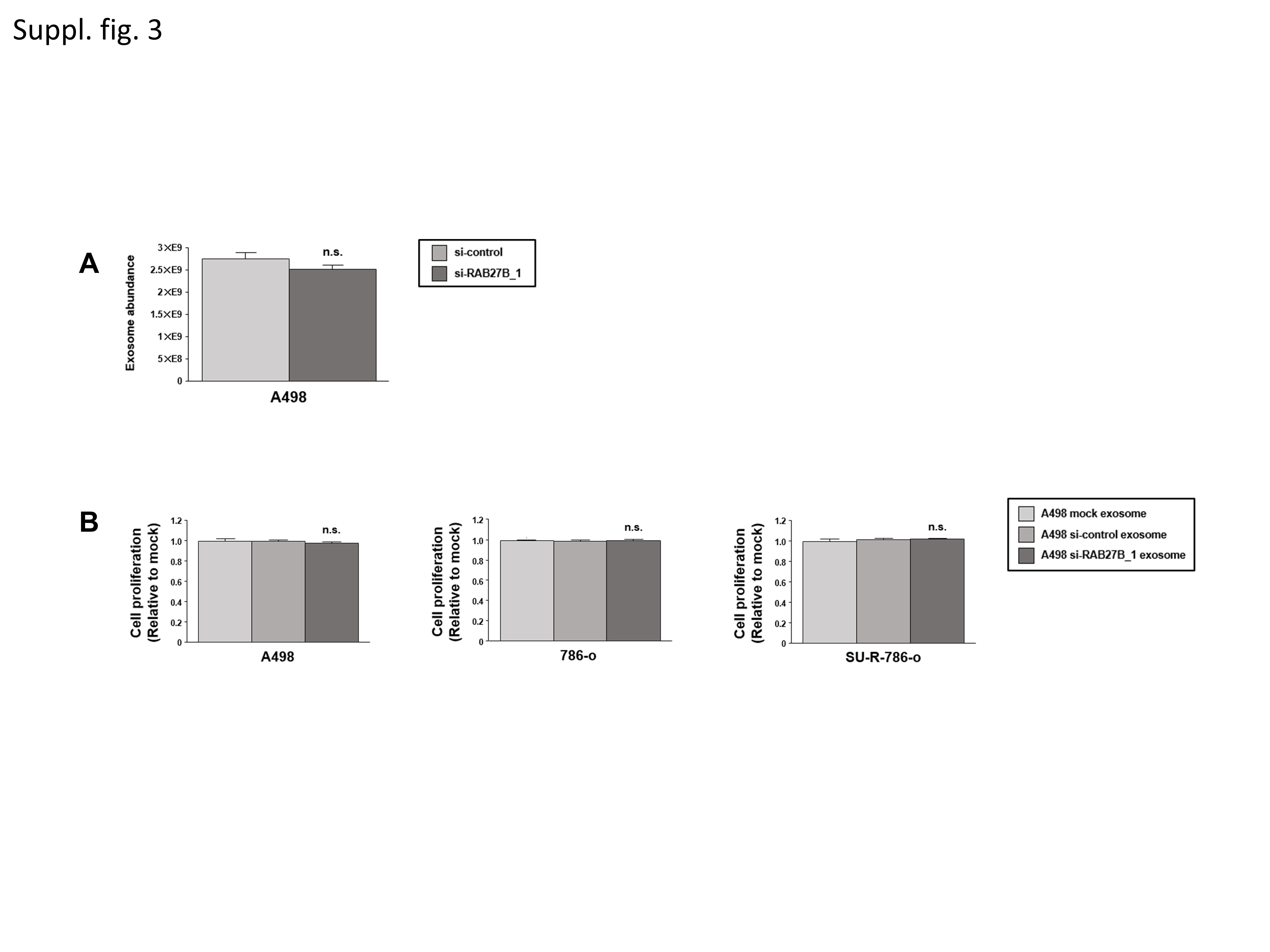

Supplement: S3 Fig — (A) Cell culture-conditioned medium was used for exosome isolation by ultracentrifugation and the amounts of exosomes were estimated by direct ELISA to CD63 on the surface of exosomes. There was no statistically significant difference between the accumulation of exosomes in si-control transfected cell culture medium and that in si-RAB27B transfected cell culture medium. (B) Exosomes derived from A498 cells were added to A498 cells (left panel), 786-o cells (middle panel) and SU-R-786-o cells (right panel). The XTT assay was performed 72 h after adding each batch of exosomes. Exosomes derived from si-RAB27B-transfected cells had no significant effect on cell proliferation compared to those from mock and si-control transfectant. n.s.; not significant. (TIF) [file pone.0232545.s003.tif]

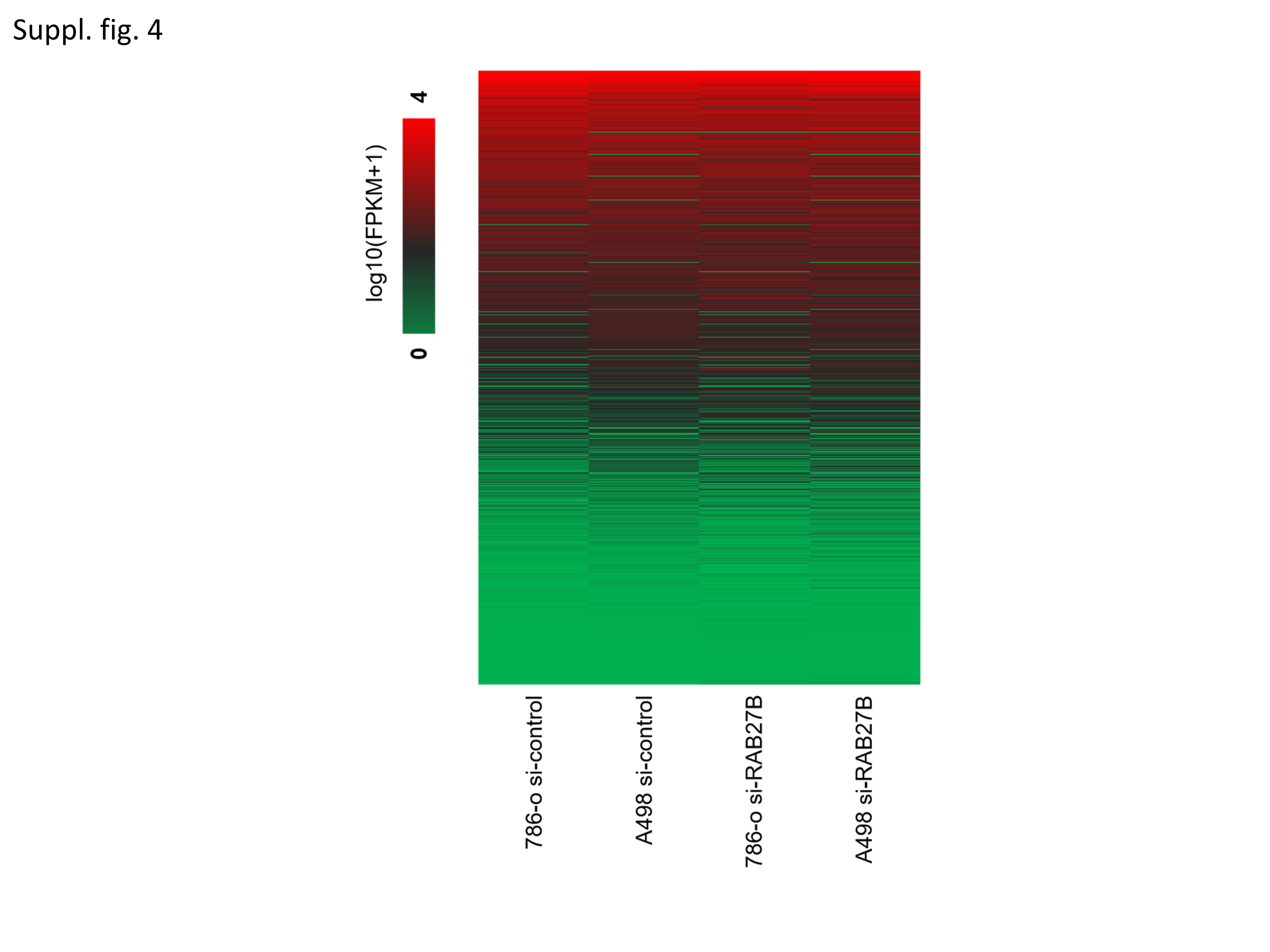

Supplement: S4 Fig — (TIF) [file pone.0232545.s004.tif]

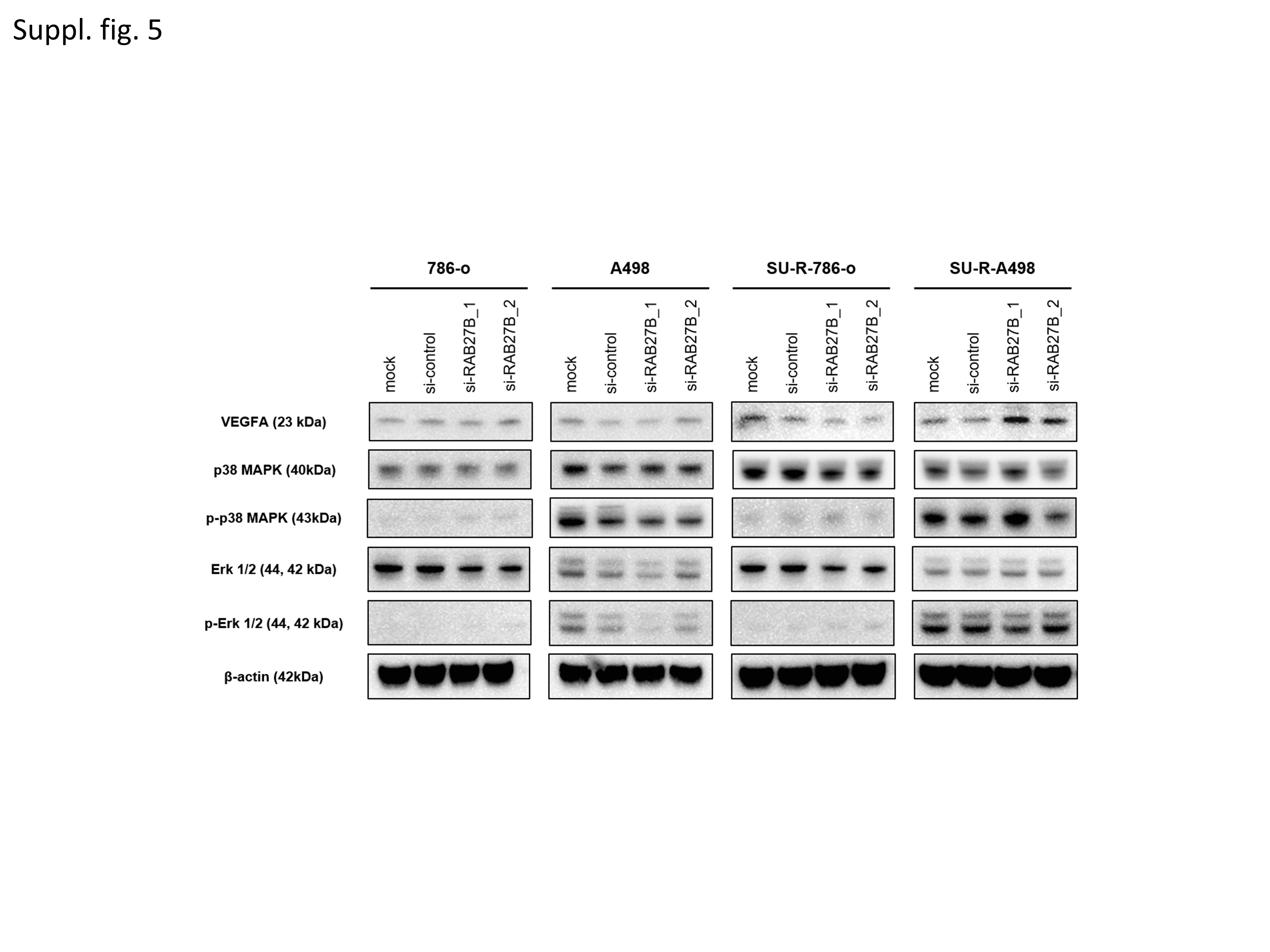

Supplement: S5 Fig — There was no certain tendency in the alteration of the expression pattern of VEGF protein after transfection of si-RAB27B. However, Erk1/2 and p38 MAPK proteins were somewhat downregulated by RAB27B knockdown in a few cell lines. (TIF) [file pone.0232545.s005.tif]

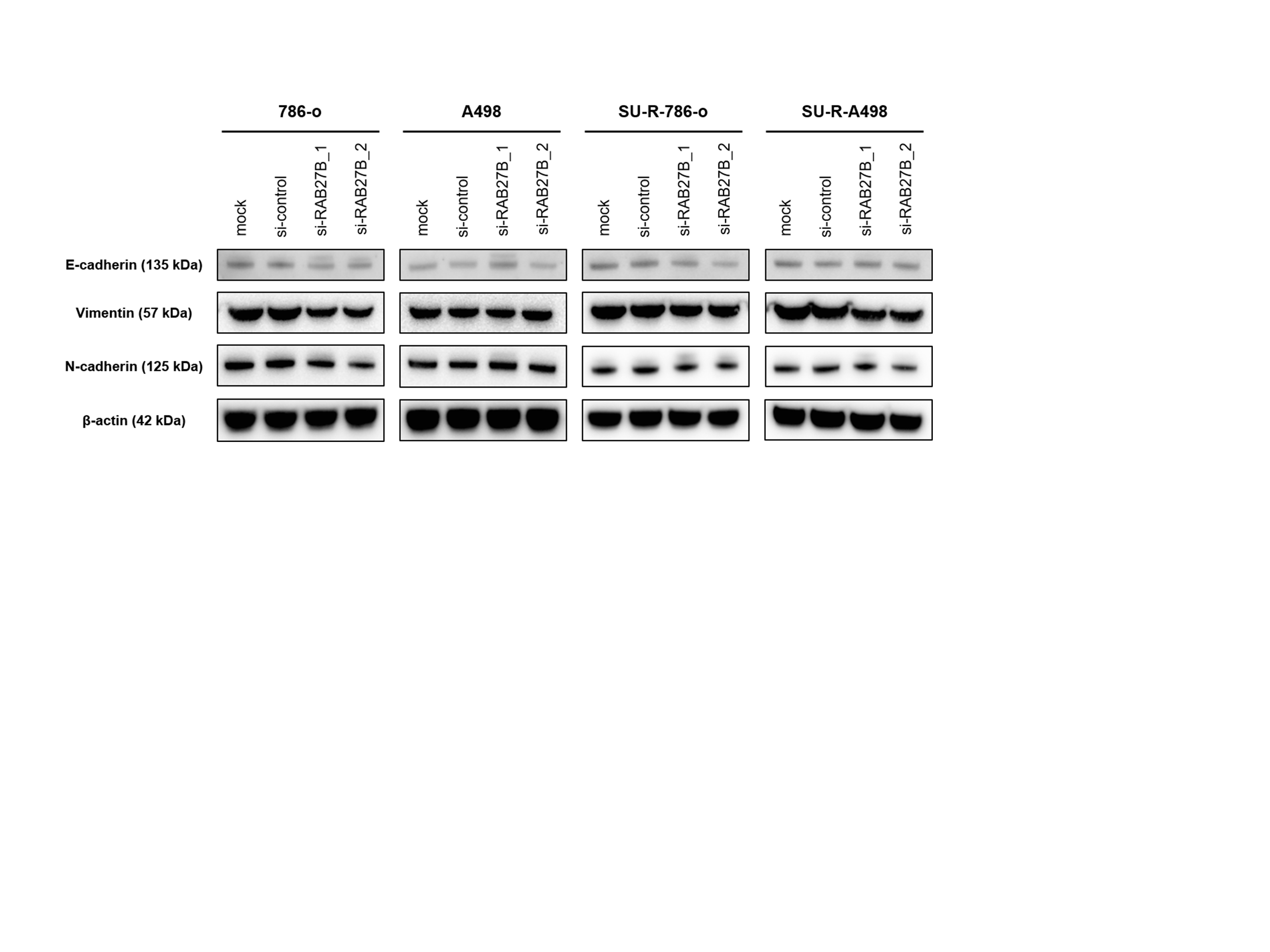

Supplement: S6 Fig — The protein expression levels of Vimentin and N-cadherin were somewhat decreased in several cell lines after knockdown of RAB27B. (TIF) [file pone.0232545.s006.tif]
